# Preanalytical Confounding Factors in the Analysis of Cerebrospinal Fluid Biomarkers for Alzheimer’s Disease: The Issue of Diurnal Variation

**DOI:** 10.3389/fneur.2015.00143

**Published:** 2015-06-29

**Authors:** Claudia Cicognola, Davide Chiasserini, Lucilla Parnetti

**Affiliations:** ^1^Section of Neurology, Department of Medicine, Centre for Memory Disturbances, University of Perugia, Perugia, Italy

**Keywords:** cerebrospinal fluid, biomarkers, diurnal variability, circadian rhythm, confounding factors, Alzheimer’s disease

## Abstract

Given the growing use of cerebrospinal fluid (CSF) beta-amyloid (Aβ) and tau as biomarkers for early diagnosis of Alzheimer’s disease (AD), it is essential that the diagnostic procedures are standardized and the results comparable across different laboratories. Preanalytical factors are reported to be the cause of at least 50% of the total variability. Among them, diurnal variability is a key issue and may have an impact on the comparability of the values obtained. The available studies on this issue are not conclusive so far. Fluctuations of CSF biomarkers in young healthy volunteers have been previously reported, while subsequent studies have not confirmed those observations in older subjects, the ones most likely to receive this test. The observed differences in circadian rhythms need to be further assessed not only in classical CSF biomarkers but also in novel forthcoming biomarkers. In this review, the existing data on the issue of diurnal variations of CSF classical biomarkers for AD will be analyzed, also evaluating the available data on new possible biomarkers.

## Relevance of CSF Biomarkers in Clinical Practice

In the past few years, the diagnostic criteria for Alzheimer’s disease (AD) have gone through several rearrangements. According to the International Working Group (IWG) for New Research Criteria for the Diagnosis of Alzheimer’s Disease, both cerebrospinal fluid (CSF) and imaging biomarkers have been recognized as mandatory for detection of AD predementia phases. The same priority has been considered in the National Institute on Aging–Alzheimer’s Association (NIA–AA) criteria (1, 2). IWG criteria subdivide clinically manifest AD in *prodromal AD* and *AD dementia*, based on whether episodic memory loss or other cognitive symptoms prevent the subject from accomplishing the instrumental activities of daily living (IADL) or not. If any AD biomarker (CSF or imaging) is abnormal, this is sufficient to fulfill the biomarker criterion for AD. The NIA-AA criteria make a distinction between amyloid markers and neuronal injury markers (tau): the likelihood of preclinical stage and MCI diagnosis is dependent on how many of the markers are positive, where amyloid is the earliest to become positive. The IWG and NIA-AA criteria share the concept of a preclinical stage of the disease, which can be recognized before dementia onset, and highlight the need of AD biomarkers both for diagnosing the disease in early stages and for supporting the diagnosis in clinically overt pathology (Table 1).

**Table 1:** **Differences between IWG and NIA-AA criteria for AD**.

Stages		Cognitive criteria		Biomarker criteria	
IWG	NIA-AA	IWG	NIA-AA	IWG	NIA-AA
Prodromal AD	MCI due to AD	Memory impairment	MCI	Any amyloid or injury marker	Likelihood: High: amyloid and injury marker both + Intermediate: one marker +, unknown the other Uninformative: one marker +, - the other
AD dementia	Dementia due to AD	Memory impairment	Dementia	Any amyloid or injury marker	

The diagnostic value of biomarkers has been even more strengthened in the IWG-2 criteria (3). In these criteria, a simplified diagnostic algorithm based on CSF molecular AD phenotype or amyloid imaging was proposed. The algorithm consisted of decreased Aβ levels together with increased t-tau or p-tau concentrations, or an increased retention on amyloid PET tracer. CSF pathophysiological markers for AD include the beta-amyloid peptide 1–42 (Aβ42), which shows lower CSF levels the more the brain carries amyloid burden, total tau (t-tau), which directly reflects the intensity of neuronal degeneration, and phosphorylated tau (p-tau), which is believed to be a direct marker of tangle pathology (4). In an autopsy cohort, low CSF Aβ42 concentrations had a sensitivity of 96.4% for AD detection (5) and CSF markers significantly increased the diagnostic accuracy in clinically uncertain cases (6). However, low CSF levels of Aβ42 are not specific enough to diagnose AD, since they can also be found in non-AD dementias (Lewy body disease or vascular dementia) (7). A valuable tool for increasing the diagnostic performance of Aβ42 is the Aβ42/40 ratio, which proved to be more reliable than Aβ42 alone in providing comprehensive information on the total Aβ load in the brain. A marked reduction in CSF Aβ42 and in the Aβ42/Aβ40 ratio has consistently been found in patients at different stages of AD (4, 8, 9), and it can help in differentiating AD from non-AD forms, where the combination of the three classical biomarkers is of limited diagnostic value (10).

Several studies have shown that the combination of CSF biomarkers may improve their global diagnostic accuracy (11–15). Data so far indicate that the combination of Aβ42 with either t-tau or p-tau has the best specificity. Additionally, the combined analysis of the CSF biomarkers provides a more accurate differential diagnosis between AD and other degenerative dementias. Aβ42 and tau (t-tau or p-tau) should be used in combination, and the simultaneous presence of low Aβ42 and high t-tau or p-tau concentrations strongly suggests an AD diagnosis even at a prodromal stage, with a sensitivity of 90–95% and a specificity of about 90% (16–20).

The importance to have reliable CSF biomarkers relies in the need to validate the clinical diagnosis with a biological correlate. Unfortunately, the results obtained in research studies are not yet totally supported by significant outcomes in routine clinical use of CSF biomarkers. Additional testing, including CSF analysis, has still little diagnostic impact in the diagnostic work-up on patients suspected to suffer AD-dementia, being rather more useful in patients with an initial non-AD dementia diagnosis (21). Reliable biomarkers are needed not only to confirm a clinical suspect but also to allow an early diagnosis, which is vital in order to prevent severe clinical manifestations by starting, as soon as possible, the disease-modifying therapies that are being developed and will be hopefully available in a near future. To this purpose, the new algorithm proposed by Lewczuk et al. (22) for diagnosing preclinical patients has further validated the diagnostic value of CSF biomarkers. The algorithm introduced the concept of “border zones” by taking into account not only the mere alteration of the biomarkers but also the extent of the alteration, from slight to clearly pathologic. This may allow the subdivision of subjects into different groups according to the CSF pattern: no evidence for CNS disease, AD improbable, AD possible, and AD probable. The results obtained with this classification may allow a better coding of the CSF patterns not clearly pathologic when classified using IWG and NIA-AA criteria. This means that the CSF profile is a valuable diagnostic tool, even in the absence of clinical symptoms.

## The Issue of Standardization of CSF AD Biomarkers for Routine Clinical Use

Even if the strong correlation between positive CSF AD biomarkers and AD pathology has been widely demonstrated, defining which patients are candidates to undergo lumbar puncture and AD CSF biomarkers analysis is a critical step for several reasons. Most of the AD patients are diagnosed using only clinical criteria, but a high number of patients do not ultimately have underlying AD pathology. The proportion of misdiagnosed patients is even higher in cases of early onset AD, atypical presentations, or dementia with mixed etiologies. It is also necessary to optimize the diagnosis of non-amnestic presentations and differentiate AD pathology from other neurodegenerative disorders, i.e., dementia with Lewy bodies, fronto-temporal dementia, vascular dementia, psychiatric conditions etc. The last consensus (2014) from the Alzheimer’s Biomarkers Standardization Initiative (ABSI) (23) focused on the issues regarding clinical use of CSF biomarkers, and stated that patients in whom AD is part of the differential diagnosis may be candidates for lumbar puncture and CSF biomarkers analysis to increase specificity and minimize diagnostic errors. Given also the importance of early diagnosis, any patient with minimal but objective symptoms suggestive of AD is an appropriate candidate. CSF biomarkers analysis should be considered in all patients with early onset dementia, minimal or mild cognitive impairment, and atypical clinical presentation or complex differential diagnosis.

With these premises, it is clear that there is a major need for standardization in the CSF analysis procedures. Standardized protocols for biobanking are a prerequisite to guarantee that biomarker studies will not be influenced by preanalytical and analytical factors. One of the most important implications of biomarker standardization is to find univocal cut-off values for CSF biomarkers between and within laboratories, given that, even when using the same assay, significant variability has been found in the absolute concentrations of AD biomarkers (24). In 2009, the Alzheimer’s Association started an international quality control (QC) program for CSF biomarkers (25). The aim of the program is to monitor, in a large network of laboratories all around the world, total analytical variability of CSF Aβ and tau, in order to identify the sources of variation and improve the standardization of the assays. All sources of variability (within-assay run, within/between-laboratory, within/between-assay kit lot) were considered, along with the variability coming from bias, systematic deviation from a reference value, imprecision and random deviation from a value. The overall variability was generally around 20–30%, with a small contribution of within-run variability (5%–10%). Within-laboratory longitudinal variability was higher, with a coefficient of variation (CV) of 5–19%. The main cause of the overall variability in the analysis of variance was the between-laboratory variability (19–28%). Even when the laboratory protocols and checklists were strictly followed, not a single factor was identified as the main source of variability. This led to the conclusion that laboratories can only be more accurate in following published guidelines (26). Moreover, it is critically important that kit manufacturers minimize lot-to-lot variations, to allow a broader use of these assays in the clinical setting. For now, the overall variability is still too high to allow the definition of univocal biomarker cut-off values; therefore, each laboratory should have internally qualified cut-off levels to guarantee optimal reproducibility over time.

### Preanalytical confounding factors of CSF biomarkers

Preanalytical factors are one of the main concerns in biochemical analysis, since they are responsible for about 40–60% of total laboratory variability (27). In previous meetings of the aforementioned ABSI, the preanalytical issues affecting Aβ and tau in CSF were discussed, and they came up with guidelines for CSF collection, storage, and analysis. Some aspects were identified as key issues for samples collection and analysis, for example, a possible CSF concentration gradient of the biomarkers. Brain-derived proteins often show a decreasing rostro-caudal gradient, implying that the volume of CSF withdrawn can alter the concentration of the proteins analyzed. Studies showed that AD CSF biomarkers concentrations are not significantly influenced by fractionated sampling, therefore gradient effect does not represent an issue in this circumstance (28). Other biomarkers can be affected, such as α-synuclein (29); therefore, a standardized volume of CSF collection (12 ml) is recommended (30). CSF for diagnostic purposes is usually obtained by lumbar puncture between the L3/L4 and L4/L5 intervertebral space, and a 22G atraumatic needle should be preferred to lower the risk of post-lumbar puncture headache (30). Moreover, a traumatic lumbar puncture increases the risk of blood contamination of the CSF sample; therefore, it is recommended to discard the first 1–2 ml to avoid any effect due to hemolysis and immediately centrifuge the sample before freezing (31). Some CSF analytes (for example, glucose) can be affected by meal consumption, making fasting a prerequisite for sampling, but this can be a problematic request in elderly patients with an AD suspect. Aβ levels in plasma proved to be stable and not influenced by the patient’s food intake (32); therefore, there is no clear evidence that meal consumption affects CSF biomarker levels and so fasting is not a requirement for the analysis. Other preanalytical confounding factors concern laboratory procedures regarding collection and storage of the samples. Aβ peptides can bind non-specifically to non-polypropylene (PP) collection tubes, leading to lower values in measured concentrations. Therefore, PP tubes are the recommended standard for CSF samples collection and testing in routine clinical practice; each laboratory should always use the same PP tube, since different tubes may have a different adsorption level for the analytes (28). Vanderstichele et al. also recommended to aliquot the samples in small volumes (0.25 or 0.5 ml tube) and fill the tube up to 75%, to minimize the risk of adsorption and evaporation (28). However, a recent study by Willemse et al. showed no evaporation of CSF stored in biobanking tubes at –80°C or –20°C over a time span of 2 years (33). As mentioned, centrifugation of CSF samples is often performed, especially in the case of hemorrhagic lumbar punctures. However, the guidelines of Vanderstichele et al. pointed out no differences in classic biomarkers levels between centrifuged versus non-centrifuged samples (29). Nevertheless, the speed and temperature of centrifugation may be considerably different across laboratories; therefore, the consensus paper by Teunissen et al. recommended to centrifuge the hemorrhagic samples at a speed of 2000 × *g* for 10 min at room temperature (26). Time and temperature of storage may have a remarkable influence on the biomarkers levels, given their effects on serum and plasma proteins showed in proteomics studies (34). Vanderstichele et al. reported no significant effects on Aβ42, t-tau, and p-tau levels when the samples are left at room temperature for 5 days after CSF collection with respect to samples frozen immediately after collection (28). The recommendation is to keep the samples at 4°C for no longer than 5 days to avoid alterations of the final biochemical results (28). Different methods of freezing and storage do not cause significant variability in the results (32), but the freezing temperature of –80°C should be preferred for long-term storage (28). Few studies have been published regarding the stability of Aβ42, t-tau, and p-tau in CSF when stored frozen at –20°C or –80°C for many years, but this is an important issue in view of longitudinal studies. Vanderstichele et al. did not observe changes in stability for up to 10 years at –80°C, but the recommendation is not to go beyond 2 months of storage at –20°C, as this is considered a sufficiently long time to run the analysis (28). The number of freeze/thaw cycles is a matter of concern since it can affect CSF biomarkers and lead to significant losses in Aβ concentrations (35). Recent studies, however, showed no significant alteration in the level of Aβ42 when CSF underwent more than one freeze/thaw cycle (32). However, freeze/thaw cycles should not be more than two and CSF must be aliquoted in small volumes; every change in the number of the freeze/thaw cycles must be accurately documented (28). The recommendations for CSF collection and storage are summarized in Table 2.

**Table 2:** **Summary of recommendations for preanalytical aspects of AD biomarker testing in CSF**.

Possible variability factor	Recommendation
CSF gradient	No gradient observed
Lumbar puncture	L3–L4 or L4–L5
	22G atraumatic needle to reduce the risk of blood contamination
Fasting	Not required
Tubes	PP (polypropylene)
Aliquotation	Aliquot the samples in small volumes (0.25 or 0.5 ml tube)
	Fill the tube up to 75%
Centrifugation	2000 × *g* for 10 min at room temperature
Time before storage	Up to 5 days
Temperature before storage	4°C
Freezing	–80°C
	–20°C for no longer than 2 months
Freeze/thaw cycles	No more than 1–2

## Circadian Rhythm

Among all the preanalytical confounding factors mentioned before, diurnal variation may play an important role as a source of variability. Circadian rhythm is involved in several physiologic processes, so it is reasonable to hypothesize its influence even in CSF biomarkers metabolism. Diurnal variation physiology must be analyzed more deeply, beginning from a review on the anatomy of this ‘‘inner clock’’ that controls a large number of bodily functions.

### Physiological aspects

The suprachiasmatic nucleus (SCN) has a central role in the circadian rhythm system, together with its three primary afferent connections (36); the most important is the retino-hypothalamic projection through which information coming from rod/cone photoreceptors and retinal ganglion cells reaches the “inner clock.” The other two afferent connections consist of the median raphe serotonergic pathway and the geniculohypothalamic (GHT) pathway from the thalamic intergeniculate leaflet (IGL). Though this network might seem elementary, the several interconnections between the pathways make it complex and convoluted. When SCN is destroyed, a wide range of bodily functions loses its daily rhythms: sleep–wake, locomotor activity, feeding, drinking, body temperature, and secretion of hormones (37). These observations were confirmed by SCN transplantation studies in which the transplant restored the lost daily rhythms (38).

One of the best-known circadian pathways is the adrenal gland axis, as glucocorticoids proved to be a humoral entraining signal for peripheral clocks (39). Rhythmic glucocorticoids release is controlled peripherally by sympathetic stimuli and centrally by the SNC, through the secretion of corticotropin releasing hormone (CRH) and ACTH (40). Behavioral processes are also under the control of the SCN, such as locomotor activity and feeding. These behaviors can be entraining factors for the “inner clock,” therefore influencing endocrine function and body temperature.

Dysfunction of circadian rhythms has been shown to have a pathogenic role in several diseases, such as cancer and autoimmune diseases. Circadian rhythm disruption may play a role not only in the etiology but also in the progression of the clinical picture. This could be a consequence of the reciprocal relationship between the neuroendocrine system and proinflammatory cytokines involved in the pathological process (41, 42). Moreover, this imbalance can act much earlier in the natural history of the disease; in fact, alterations in sleeping and eating patterns in humans were found to be a source of predisposition to metabolic and cardiovascular diseases (41). A diurnal variation of the symptoms is also typical of many diseases with an immune or inflammatory component. For example, in rheumatoid arthritis, patients refer more joint pain and stiffness in the morning hours, whereas patients with osteoarthritis refer a pain that increases through the day (41).

### Circadian rhythms in AD

Many of the physiological bodily functions described become impaired in AD, but also in other neurodegenerative disorders such as Parkinson’s disease and Huntington’s disease. In these conditions, several brain areas are affected by neurodegenerative processes, including the nuclei involved in circadian regulation. Neurodegenerative disorders are associated with several sleep–wake rhythm disturbances, such as insomnia/hypersomnia, parasomnia, excessive nocturnal motor activity (for example, restless legs syndrome), and sleep apnea. In AD, sleep is often irregular and disturbed by multiple awakenings and, along with disease duration and progression to advanced stage, a phase shift of the sleep period is observed, often leading to a complete reversal of the day/night pattern (43). These signs and symptoms not only contribute to morbidity, poor quality of life, and institutionalization of individuals with AD (44) but could also be involved in the etiology of the pathological process (45).

Changes in rest-activity patterns correlate with the severity of dementia and could be a preclinical marker, in healthy subjects, of predisposition and possible future development of cognitive impairment and AD (46). A prospective actigraphy study led in a cohort of 1282 healthy women showed higher incidence of MCI and dementia in women with decreased circadian activity rhythm amplitude at follow up (approximately 5 years later). Reductions in total melatonin (the molecule that controls night-day cycle) levels are more profound in AD than in normally aging individuals, as showed in a post-mortem study (47–49). Melatonin showed protective anti-amyloidogenic effects *in vitro* and, interestingly, was found to be decreased in early (even preclinical) stages of the disease, both in total levels and width of the circadian oscillations (49, 50). These findings were supported by the observation of a decrease in the number of melatonin receptor-carrying neurons in the SCN in late-stage AD, alongside with a decrease of volume and total cell count in the whole SCN itself (51, 52). Moreover, the expression of “clock genes” is altered in the brain of AD patients, reflecting the disruption of the master control by the SCN (53). These alterations in circadian rhythms were demonstrated in animal models transgenic for AD-associated mutations (54).

Circadian disruption can be both a consequence of AD as well as worsening factor in AD pathological cascade, suggesting a biunivocal relationship between the two (55). On one hand, AD pathology can lead to day/night sleep pattern disturbances and subsequent poor quality of life; on the other hand, the same disturbances can influence the course of AD pathology. Sleep deprivation results in increased concentration and accumulation of Aβ, in contrast to sleep extension that has the opposite effect. The accumulation of Aβ results in increased wakefulness and altered sleep pattern, as observed in sleep-restricted animals that showed greater Aβ plaque deposition compared to controls (56). Studies on orexin, also known as hypocretin, (a molecule that regulates wakefulness, strongly implicated in sleep disorders) showed that its release from hypothalamic neurons and the pattern of Aβ in CSF have a comparable diurnal fluctuation, and that orexin itself shows a circadian rhythm in both AD patients and controls (56, 57). Aβ levels are also increased during orexin infusion and decreased with an orexin receptor antagonist, indicating a role of orexin and sleep-wake cycle disruption in the pathogenesis of AD (52). Low CSF Aβ42 levels have been found to be related to lower levels of orexin, further suggesting a relationship between AD pathology and orexin disturbance (57, 58). A clinical trial on a population of healthy middle-aged men confirmed these observations, showing a decrease of 6% in the CSF Aβ42 levels after one night of unrestricted sleep and a difference of 75.8 pg/ml between the CSF Aβ42 levels of the unrestricted sleep and sleep deprived group (59).

## Diurnal Variation of CSF AD Biomarkers: State of the Art

As previously reported, diurnal variation can be a critical factor while studying molecules that can be influenced by circadian rhythms, making sampling time a matter of concern. Focusing on AD CSF biomarkers, Bateman et al. showed that human CSF Aβ levels varied significantly (1.5- to 4-fold) over 36 h (60). The Aβ levels showed no significant differences between the hours during the daytime period, but an increase during a 36-h period. All participants were screened to be in good general health and without neurologic diseases. Participants older than 65 were non-demented controls, and had a Clinical Dementia Rating of 0. Six milliliters of CSF were obtained each hour for 12, 24, or 36 h. CSF aliquots were frozen at –80°C immediately after collection in 1 ml PP tubes. One milliliter of CSF from each collection hour was thawed and Aβ40 and Aβ42 were measured by ELISA. A sinusoidal pattern of Aβ levels was observed across participants, supposed to be due to time of day, activity, or dynamic changes in the production or clearance rate of Aβ in the CNS. The study by Bateman was the first to arise the issue of a possible diurnal variation of CSF biomarkers that could represent a significant obstacle to an accurate diagnosis. However, a previous study by Andreasen et al. showed no significant fluctuations of Aβ42 on repeated lumbar puncture in subjects with AD (61). It may be that CSF Aβ variability is decreased in patients with AD pathology and amyloid plaques, but has higher fluctuations in individuals without plaques. Bjerke et al. also found no diurnal variation in 14 psychiatrically and neurologically healthy subjects carrying lumbar catheters due to knee surgery (32); CSF was serially collected by lumbar puncture at baseline, after 4–6 h and after 24 h. The samples were immediately stored at –80°C. Data showed more stable levels with a slight but significant decrease in CSF Aβ42 after 4–6 h, which tended to return to baseline levels after 24 h. A possible reason for these results is that, as opposed to Bateman et al., a smaller CSF volume was taken; this could have led to a minor impact on the CSF dynamics. Slats et al. also found no diurnal variation in CSF dynamics during a 36-h sampling (6 ml per hour) (62). They investigated the within-subject variability over 36 h in CSF Aβ and tau proteins, in older subjects, and AD patients. Six patients with mild stage AD [59–85 years, mini mental state examination (MMSE) 16–26 range] and six healthy older volunteers (64–77 years) underwent insertion of an intrathecal catheter from which 6 ml of CSF were collected each hour for 36 h. Variability of CSF Aβ40, Aβ42, t-tau, and p-tau concentrations was lower than expected and the diurnal variation was not as wide as in the younger subjects in Bateman’s study. The most recent study was led by Moghekar et al. in a cohort of older mildly symptomatic individuals to determine whether CSF biomarkers of AD fluctuate significantly over time (63). Ten patients suspected of having idiopathic normal pressure hydrocephalus or pseudotumor cerebri were recruited. Intracranial pressure monitoring and CSF drainage represented part of their routine clinical care. Most of the patients had relatively modest cognitive problems associated with their suspected diagnosis (MMSE score range 20–30). Clinical diagnoses of dementia and MCI were based on informant history as well as cognitive testing, without knowledge of AD biomarker levels. All patients underwent insertion of a catheter into the lumbar subarachnoid space on the first day of hospitalization. After monitoring of intracranial pressure for 18 h, drainage of CSF was initiated at noon the following day. Collection of CSF for analysis started at 6 p.m. on the first day of drainage (the second hospital day). Forty milliliters of CSF were withdrawn from the lumbar catheter every 6 h for 24 or 36 consecutive hours and then stored at –80°C until further analysis. The levels of Aβ42, Aβ40, total tau, and p-tau, although significantly different between the patients, did not fluctuate appreciably over time. Significant fluctuations in Aβ did not occur in the patients with the highest CSF Aβ levels as well as in those with the lowest CSF Aβ levels. This study and the one from Bateman et al. have two major differences: age and health status of the population and sampling frequency. Population was significantly older and with ongoing neurological abnormalities, opposed to the young healthy subjects of Bateman’s study; still, the role of age is uncertain, since no great differences were found in the fluctuations of Aβ42 between the youngest and oldest patients in the cohort. The samples were collected every 6 h instead of each hour as in Bateman’s study; however, since the peak-to-peak variability for Aβ followed a 12-h cycle in the prior study, a significant level of variability would have been apparent in the latter study. All the results are summarized in Table 3.

**Table 3:** **Summary of the effects of diurnal variation on CSF AD biomarkers levels**.

Reference #	Demographics	Population size	Samples collection	Assay	Effects of diurnal variation
(60)	Non-demented subjects: 23–78 years old	15	6 ml CSF from lumbar catheter Each hour for 12, 24, or 36 h CSF frozen at –80°C after collection in polypropylene tubes	Aβ determined with ELISA	Aβ varied significantly, showing an increase over a 36 h period
(32)	Healthy subjects undergoing knee surgery	14	10–12 ml CSF from lumbar puncture Baseline, after 4–6 h, after 24 h CSF frozen at –80°C after collection in polypropylene tubes	Aβ42 determined with xMAP-based assay	No significant diurnal variation, slight decrease in Aβ42 levels that tended to return to baseline after 24 h
(62)	Mild stage AD patients: 59–85 years old; healthy volunteers: 64–77 years old	6 + 6	6 ml CSF from intrathecal catheter During 36 h, each hour CSF frozen at –80°C after collection in polypropylene tubes	Aβ42, t-tau, and p-tau determined with xMAP-based assay. Aβ40 determined with ELISA	No significant diurnal variation, less pronounced circadian pattern compared with the one in younger subjects
(63)	Patients suspected of having idiopathic normal pressure hydrocephalus (*n* = 9) or pseudotumor cerebri (*n* = 1)	10	40 ml CSF from lumbar catheter Every 6 h for 24 or 36 consecutive hours CSF frozen at –80°C after collection in polypropylene tubes	Aβ42, total tau, and p-tau181 determined with xMAP-based assay. Aβ40 determined with ELISA	No significant diurnal variation

Amyloid metabolism is characterized by several critical steps, which can cause variability in its CSF levels: production from cleavage of amyloid precursor protein (APP), degradation by proteases and microglia, and clearance by systemic circulation or lymphatics (64). However, up to now, none of these steps justifies the diurnal fluctuations of Aβ reported by Bateman, except for the diurnal variation in transcription, translation of APP, and regulation of the two secretases (beta or gamma secretase) that cleave APP to produce Aβ (65). In CNS, APP can be cleaved by either the β-secretase pathway or the α-secretase pathway: the first is amyloidogenic and generates soluble APP-β (sAPPβ) and Aβ; the second one is non-amyloidogenic and causes the release of soluble APP-α (sAPPα). In 2014, Dobrowolska et al. measured APP proteolytic products over 36 h in the CSF of cognitively normal and AD individuals, in order to clarify the role of APP metabolism in α- and β- pathway balance and, consequently, in Aβ diurnal pattern. Diurnal fluctuations were found in sAPPα, sAPPβ, Aβ40, and Aβ42, diminishing with increased age; these findings support the hypothesis that APP undergoes a circadian rhythm regulated in the central nervous system and thus results in Aβ diurnal fluctuations. Moreover, it was found that the ratio of sAPPβ to sAPPα was significantly higher in participants with cerebral Aβ deposits compared to those without deposits, therefore making sAPPβ/sAPPα ratio a valuable biomarker for cerebral amyloidosis (65). Time-dependent fluctuations were also observed in CSF Apolipoprotein E (apoE) (66, 67). ApoE is the probably the most important and acknowledged genetic risk factor for Alzheimer’s disease and even for some other neurological disorders. ApoE has several isoforms; the most common are represented by ApoE2 and E4, each associated with a different effect on AD predisposition, with ApoE4 increasing and ApoE2 decreasing the risk of AD. Their role in modulating AD pathology is due to both the isoform and amount of ApoE in the brain, which directly reflects the extent of Aβ peptide deposition. Therefore, quantifying ApoE isoforms, especially ApoE4, could be a useful biological correlate in the study of AD pathology from preclinical to clinical stages. The main obstacle to the use of ApoE4 as a biomarker is that CNS and peripheral ApoE isoform turnover rates differ substantially, probably because the ApoE metabolism pathways are different in the CNS and the periphery, as observed in a study by Wildsmith et al. (67). The study also showed different turnover rates for each isoform of ApoE and a slower turnover rate for CSF ApoE than the Aβ one. Further *in vivo* studies are needed to determine if the fluctuations in ApoE metabolism are a limiting factor for its use as biomarker.

## Conclusion

Classical AD CSF biomarkers show a promising diagnostic value, especially in research studies; current and future research should be devoted to the standardization of the collection and analysis procedures to increase the statistical power of the results and the comparability among laboratories. Univocal cut-off values for biomarkers should be available, along with standard operating procedures for preanalytical factors. The aim is to allow a more widespread use of CSF biomarkers and lumbar puncture in clinical routine for early AD diagnosis.

It must also be taken into account that most studies on diurnal variation focus only on Aβ and do not consider other neuronal injury markers. Up to now there are very few studies focusing on tau-related biomarkers and their possible fluctuations during the daytime period (62, 63). When speaking of accuracy of CSF diagnostics, further studies need to be performed before ruling out diurnal variation as a variability factor. Nevertheless, data so far are reassuring, since no significant diurnal fluctuations have been consistently found.

In case of novel biomarkers, the suggestion is that diurnal variation always needs to be analyzed as a variability factor; the recommendation is to record withdrawal time information to identify the possible diurnal variation of the new analyte. In conclusion, taking into account that CSF withdrawal is usually performed during daytime and that no significant changes have been found in the levels of classic AD CSF biomarkers at different times of day, there is no need to standardize a specific time interval during the day for CSF collection.

## Conflict of Interest Statement

The authors declare that the research was conducted in the absence of any commercial or financial relationships that could be construed as a potential conflict of interest.
